# Women with chronic pelvic pain can be stratified using multimodal assessment

**DOI:** 10.1097/j.pain.0000000000003857

**Published:** 2025-11-17

**Authors:** Lysia Demetriou, Lydia Coxon, Emma Evans, Kevin Kuan, Danielle Perro, Kirsten Parsons, Emily Tan, Ana Charrua, Joana Ferreira-Gomes, Pedro Abreu Mendes, Claire E. Lunde, Lars Arendt-Nielsen, Qasim Aziz, Judy Birch, Kurtis Garbutt, Anja Hoffman, Andrew W. Horne, Andreas Schilder, Lone Hummelshoj, Michal Krassowski, Jane Meijlink, Esther M. Pogatzki-Zahn, Rolf-Detlef Treede, Allison F. Vitonis, Jan Vollert, Christian M. Becker, Francisco Cruz, Stacey A. Missmer, Christine B. Sieberg, Krina Zondervan, Jens Nagel, Katy Vincent

**Affiliations:** a Nuffield Department of Women's and Reproductive Health, University of Oxford, Oxford, United Kingdom; b Department of Obstetrics and Gynaecology, University of Toronto, Toronto, ON, Canada; c Edinburgh Medical School, University of Edinburgh, Edinburgh, Scotland; d I3S—Instituto de Investigação e Inovação em Saúde, Universidade do Porto, Porto, Portugal; e Departamento de Biomedicina—Unidade de Biologia Experimental, Faculdade de Medicina da Universidade do Porto, Porto, Portugal; f RISE Health, Department of Biomedicine, Faculty of Medicine, University of Porto, Porto, Portugal; g Biobehavioral Pain Innovations Lab, Department of Psychiatry and Behavioral Sciences, Boston Children's Hospital, Boston, MA, United States; h Department of Anesthesiology, Critical Care, and Pain Medicine, Pain and Affective Neuroscience Center, Boston Children's Hospital, Boston, MA, United States; i Department of Health Science and Technology, Center for Neuroplasticity and Pain (CNAP), SMI, School of Medicine, Aalborg University, Aalborg, Denmark; j Department of Medical Gastroenterology, Mech-Sense, Aalborg UniversityHospital, Aalborg, Denmark; k Steno Diabetes Center North Denmark, Clinical Institute, Aalborg UniversityHospital, Aalborg, Denmark; l Centre for Neuroscience, Surgery and Trauma, Blizard Institute, Wingate Institute of Neurogastroenterology, Barts and The London School of Medicine and Dentistry, London, United Kingdom; m Pelvic Pain Support Network, Poole, United Kingdom; n Research and Development, Pharmaceuticals Experimental Medicine, Bayer AG, Berlin, Germany; o Centre for Reproductive Health, University of Edinburgh, Edinburgh, United Kingdom; p Department of Orthopaedic and Trauma Surgery, University Medical Centre Mannheim, Medical Faculty Mannheim, University of Heidelberg, Mannheim, Germany; q Endometriosis.org, London, United Kingdom; r International Painful Bladder Foundation, Naarden, the Netherlands; s Department of Anaesthesiology, Intensive Care and Pain Medicine, University Hospital Muenster, Muenster, Germany; t Department of Neurophysiology, Department of Psychiatry and Psychotehrapy, Medical Faculty Mannheim, Heidelberg University, Mannheim, Germany; u Department of Obstetrics and Gynecology, Brigham and Women's Hospital and Harvard Medical School, Boston, MA, United States; v Boston Center for Endometriosis, Boston Children's Hospital and Brigham and Women's Hospital, Boston, MA, United States; w Department of Clinical and Biomedical Sciences, Faculty of Health and Life Sciences, University of Exeter, Exeter, United Kingdom; x Department of Obstetrics, Gynecology, and Reproductive Biology, College of Human Medicine, Michigan State University, Grand Rapids, MI, United States; y Division of Adolescent and Young Adult Medicine, Department of Pediatrics, Boston Children's Hospital, Harvard Medical School, Boston, MA, United States; z Department of Epidemiology, Harvard T.H. Chan School of Public Health, Boston, MA, United States; aa Departamento de Biomedicina – Unidade de Biologia Experimental, Faculdade de Medicina da Universidade do Porto, Porto, Portugal; ab Department of Psychiatry, Harvard Medical School, Boston, MA, United States; ac Center for Health Outcomes & Interdisciplinary Research, Department of Psychiatry, Mass General Hospital, Boston, MA, United States; ad Exploratory Pathobiology, Research and Development, Pharmaceuticals, Bayer Aktiengesellschaft, Wuppertal, Germany.

**Keywords:** Chronic pelvic pain, Endometriosis, Bladder pain, Latent profile analysis, Multimodal assessment

## Abstract

Chronic pelvic pain (CPP) is a common and burdensome symptom in women yet current clinical management frequently leaves many with persistent pain. The Translational Research in Pelvic Pain (TRiPP) project adopts a pain-focused strategy, aiming to better phenotype CPP through multimodal assessment. Here, we integrated questionnaire, physiological, and biological data to (1) determine whether perturbations in the function of pain-relevant systems in women with CPP can be demonstrated and (2) explore whether these data can stratify women with CPP into meaningful subgroups, independent of diagnostic group. Participants included 108 women, aged 18 to 50 years, with CPP including endometriosis-associated pain (EAP), bladder pain syndrome (BPS), comorbid EAP and BPS, and pelvic pain with no underlying pathology alongside 50 pain-free controls. Analyses were conducted in 3 stages: (1) group comparisons, (2) latent profile analysis to identify CPP subgroups, and (3) clinical characterization of resulting clusters. Compared with controls, CPP participants reported significantly greater fatigue, poorer sleep, higher anxiety, depression, pain catastrophising, and more childhood trauma (*P* < 0.01). However, no significant differences were observed in physiological measures. Latent profile analysis revealed 3 distinct CPP subgroups, differentiated by questionnaires rather than physiological measures. Cluster 1 represents a group with predominantly higher-impact pain compared with others, that may be associated with nociplastic mechanisms. These findings support alternative approaches to stratification of CPP separate from standard diagnostic groupings. The role of questionnaire measures in this stratification facilitates translation to clinical settings; however, further work is required to determine whether differing therapeutic approaches are appropriate for each cluster.

## Abstract 2

Supplemental Digital Content is Available in the Text.

Women with chronic pelvic pain can be stratified into distinct subgroups using multimodal assessment, supporting personalized, pain-focused therapeutic strategies beyond traditional pelvic diagnoses.

## 1. Introduction

Globally, chronic pelvic pain (CPP) affects up to 26.6% of women and those born female,^3,14,51,52^ affecting quality of life and incurring substantial healthcare and economic costs.^25^ Despite the high prevalence, underlying mechanisms remain poorly understood, and existing management strategies are unsatisfactory.

Chronic pelvic pain is classified as a secondary pain condition if associated with an underlying pathology such as endometriosis (International Classidication of Diseases [ICD-11], Code: MG30),^47^ with therapeutic approaches focussing on the pathology. For those without identifiable pathology, symptom constellations determine the appropriate primary pain condition, eg, interstitial cystitis/bladder pain syndrome (IC/BPS). Whether primary or secondary, patients with CPP are predominantly seen by the clinicians responsible for the pelvic organ(s) considered most likely the cause of their pain, eg, gynaecologists, urologists, and gastroenterologists. Despite increasing evidence of similarities with other chronic pain conditions, a pain-focussed approach usually comes late in the journey (if at all) for these women.^34^

Women with CPP commonly describe many years of pain, which frequently persists or recurs despite recommended treatment of any identified associated pathology.^7,8,23,34^ Thus, there is an urgent need to identify alternative approaches for stratifying those with CPP into clinically meaningful groups and to define appropriate treatment algorithms for these groups. Work to date has explored possible approaches for subgrouping those with specific types of CPP, including IC/BPS,^22,37,49^ vulvodynia,^4,21,41^ and endometriosis-associated pain,^27^ and for CPP more generally.^6^ These approaches seem to be able to identify subgroups with high-impact pain and, in IC/BPS, to impact on treatment response.^17^ These studies have predominantly used questionnaire measures, or where clinical/psychophysical assessments have been used, these have focussed on the pelvis (eg, bladder capacity; sensitivity of the bladder to filling, the pelvic floor muscles to pressure, and the vulva to experimental stimuli). Given increasing awareness that factors outside the pelvis are of importance in predicting response to treatment,^5,17^ it is plausible that taking a broader approach, as has been performed for other types of chronic pain,^15,16^ may give greater insights. However, relatively little is known about the responses of those with CPP to many pain-relevant psychophysical assessments.

To address this knowledge gap, in this article, we use data from the Translational Research in Pelvic Pain (TRiPP) project (https://www.imi-paincare.eu/PROJECT/TRIPP/), a project which aimed to take a deep-phenotyping approach to improve understanding of CPP in women, including better methods of stratification.^11^ We aimed first to determine whether we can demonstrate perturbations in the function of pain-relevant systems in women with CPP compared with pain-free women and, second, to explore whether we can use these data to stratify women with CPP into meaningful subgroups. We hypothesised that at a group level (comparing those with CPP with pain-free controls), we would see differences in the assessed measures. However, we expected that there would be significant heterogeneity in all measures for those with CPP and that this variation would allow the identification of subgroups using a data-driven approach. A main hypothesis of our consortium was that these subgroups may be independent from the diagnostic label.

## 2. Methods

### 2.1. Participant recruitment

Participant recruitment for the TRiPP project took place across 3 sites: University of Oxford, UK; Boston's Children's Hospital, USA; and Instituto de Biologia Molecular e Celular, Portugal. Participants were women of reproductive age (18-50 years) with and without chronic pelvic pain. The CPP group comprised 4 pelvic pain sub-groups with different underlying diagnoses: (1) Endometriosis-associated pain (EAP): individuals with a prior surgical diagnosis of endometriosis and at least 1 type of pelvic pain rated >4/10; (2) Endometriosis-associated pain with comorbid bladder pain (EABP): participants meeting EAP criteria with additional bladder-related pain and urinary symptoms (frequency and/or urgency); (3) BPS: individuals reporting bladder pain >4/10 and urinary symptoms (frequency and/or urgency) without a prior surgical diagnosis of endometriosis; and (4) Pelvic pain without bladder or urinary symptoms and no prior endometriosis diagnosis (PP). All CPP participants had reported at least 1 pelvic pain score of >4/10. Participants in the control group had no history of endometriosis, no urinary symptoms, and reported pelvic pain of <3/10 on a numerical rating scale (NRS). The full protocol and selection criteria for the cohort are described in the study protocol (Demetriou et al., 2022).

### 2.2. Study data

#### 2.2.1. Questionnaire data

Chronic pelvic pain participants were asked to complete a set of questionnaires selected to capture a full picture of their pain experience, other potentially relevant clinical variables, quality of life, pain interference, and other measures previously described as being relevant to visceral pain summarized in Table 1. Pain-free controls completed all the same measures except those capturing additional detail about their pelvic pain (eg, the painDETECT scale).

**Table 1:** Summary of assessment domains, measurement tools with scoring procedures, and interpretation guidelines for all clinical, psychological, and physiological variables.

Domain	Tool	Scoring	Interpretation of measure
Pain			
Intensity	Numerical rating scale (NRS) on Worst pelvic pain in the past 3 mo (noncyclical pelvic pain) Pain during their last period (dysmenorrhea) Worst bladder pain in the past week Pain during sexual intercourse (dyspareunia) 24 h after sexual intercourse (dyspareunia)	Average of score	0 (“no pain”)-10 (“worst imaginable pain”)
Nature	painDetect	Scored as per the Freynhagen et al. Instructions	0-19 (higher scores indicated a higher likelihood of neuropathic pain)
Widespreadness	Fibromyalgia survey scale	Pain widespreadness calculated using the Michigan body map to determine the number of extra-pelvic regions which the participants marked as having felt persistent or recurrent pain in the past 3 mo or longer	0-32 (number of regions of pain)
Pain interference	Scale from EPHect clinical covariates questionnaire	Scale assessing the extend pain interfered with normal social activities in the past 3 mo: work/school, daily activities at home, sleep, sexual intercourse, exercise/sports, social activities	Frequency of pain interreference on a nominal scale: (1) not at all, (2) slightly, (3) moderately, (4) quite a bit, and (5) extremely
Comorbidities	Self-report of diagnosis received by a clinician	Frequency of diagnosis	
Gastrointestinal symptoms	The gastrointestinal symptom rating scale	Average score for each a total GSRS score and for each subscale: reflux, abdominal pain, indigestion, diarrhoea, and constipation. As per the Revicki 1997 guidelines	0-7 (higher scores indicate higher symptom discomfort)
Mood	Hospital anxiety and depression scale (HADS)	Sum of scores for each subscale (anxiety and depression)	0-21 (higher scores indicated higher levels of depression or anxiety)
Pain catastrophising	Pain catastrophising scale	Sum of scores for the total PCS and each subscale: rumination, magnification, and helplessness	0-52 (higher score indicates higher levels of catastrophising)
Quality of life (QoL)	Short form health survey (SF-36)	Average of scores for each subscale: physical functioning, role limitations due to physical health, role limitations due to emotional problems, energy/fatigue, emotional wellbeing, social functioning, pain, general health (scored as per the validated scoring system by Rand Corporation [https://www.rand.org/health-care/surveys_tools/mos/36-item-short-form/scoring.html] and Burholt et al.)	0-100 (higher scores indicate better QoL)
Personality	The Big 5 personality inventory	Average score for each of the 5 key dimensions of personality: Openness (creativity and open-mindedness), conscientiousness (organization and dependability), extraversion (sociability and assertiveness), agreeableness (compassion and cooperativeness), and neuroticism (emotional stability and stress levels)	1-5 (higher score indicate higher levels of the specific trait, ie, being measured)
Fatigue	Neuro-QOL v1 fatigue	t-scores were calculated following guidelines of the Health Measures Scoring Service	20-80 (higher scores indicate higher fatigue levels)
Sleep	ASCQ-Me v2 sleep impact short form	t-scores were calculated following guidelines of the Health Measures Scoring Service	20-80 (higher scores indicate lower sleep quality)
Trauma	(1) Childhood traumatic events scale (2) Recent traumatic events scale	The number and burden of trauma was calculated for each scale	Higher number indicates more traumatic experiences or burden
Somatosensory processing	QST on the lower abdomen (test site) and dorsum of the foot (control site)		
Visceral sensitivity	Bladder sensitivity paradigm	Assessed pain intensity at first sensation and first urge	
Autonomic nervous system function	Heart rate	Heart rate monitored pre and post the CPM pain paradigm	
HPA axis function	(1) 24-hour cortisol profile (saliva) (2) Cortisol before and after pain stimuli (saliva)	Measurement of cortisol concentrations (nmol/L) (1) Area under the curve was calculated based on the 24-hour measurements (2) Cortisol response was calculated as (postcortisol − precortisol levels) before and after the pain paradigm	
Endogenous pain modulation	Conditioned pain modulation paradigm		

#### 2.2.2. Physiological and biological measures

##### 2.2.2.1. Electrocardiogram

Twenty minutes of a 12 lead electrocardiogram (ECG) was conducted^12^ at rest, lying down, before and after the CPM paradigm (described below) to assess the heart rate.

##### 2.2.2.2. Cortisol

A 24-hour profile of cortisol levels was assessed using saliva samples. Participants were asked to provide 5 saliva samples using a saliva kit during a normal day (in which no study testing took place): (1) as soon as they wake-up, (2) 30 to 45 minutes after waking up, (3) before lunch, (4) before dinner, and (5) bedtime. They were asked to record the exact time of each sample and store them in the fridge until bringing them back to the researchers for processing. In addition, saliva samples were collected pre and post physiological testing.

##### 2.2.2.3. Quantitative sensory testing

The German Neuropathic Pain Network quantitative sensory testing (QST) paradigm^36,45^ was conducted by trained team members. These tests include assessment of thermal and mechanical detection and pain thresholds, as well as vibration detection, mechanical sensitivity, wind-up ratio, and pressure pain thresholds, giving an overall 13 measures. We conducted testing on the dorsum of the right foot (control site) and lower abdomen/pelvis below the umbilicus (test site). All testing were conducted in a temperature-controlled room at approximately 20°C. For participants from the Institute of Molecular and Cell Biology (IBMC), testing was conducted in Portuguese, with the script forward and backward translated for accuracy. Full description of the paradigm, analysis, and results is published in Coxon et al., (2023).^9^

##### 2.2.2.4. Conditioned pain modulation

The conditioned pain modulation (CPM) paradigm assesses the efficiency of the body's endogenous pain inhibitory pathways. Participants underwent CPM testing in a temperature-controlled room (20°C). Pressure pain thresholds (PPT) were assessed using a force dial algometer applied 3 times to the right dorsal foot, with the mean pressure (PPT1_average_) recorded as baseline. A pressure cuff conditioning stimulus (CS) was then applied to the left arm, inflated until participants reported pain, and maintained for 60 seconds. Before deflation, participants rated their pain (0-10), and the CS pressure was recorded. Immediately after, the algometer was reapplied to the foot to determine PPT2_average_. After a 10-minute rest, the procedure was repeated, recording the CS pain rating and PPT3_average_ to assess CPM effects. A full description of the CPM paradigm and analysis is provided in Demetriou et al., 2025.^12^

##### 2.2.2.5. Bladder paradigm

We used a noninvasive bladder paradigm in this study, which has been previously developed.^48^ Participants are asked first to void their bladder and then to drink 20 fl.oz (US) of water in 5 minutes. They are instructed to inform the researcher when they reach certain sensations, at which point in time (since onset of drinking), pain intensity rating (NRS 0 [no pain]—10 [worst pain imaginable]) and urgency rating (NRS 0 [no urgency]—10 [worst urgency imaginable]) are recorded. These sensations are First Sensation (described as “when riding in a car, the drivers pulls over to a rest-stop to urinate, you would go as well”), First Urge (“when riding in a car, you would initiate the request to find a rest-stop to urinate”), and Maximum Tolerance (“when riding in a car, you would urinate on the side of the road in bumper-to-bumper traffic”). Once participants have reached Maximum Tolerance (or after 2 hours, whichever occurs first), the participant is asked to void, and the volume of urine is recorded by the researcher.

### 2.3. Data analysis

**Figure 1.**
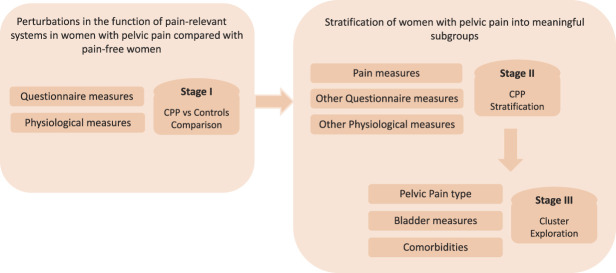
Overview of the study aims and analysis pipeline.

As illustrated in Figure 1, our approach to analysis was in 3 stages. First (stage 1), to address aim 1, we compared those with CPP with the pain-free control group. For these analyses, we used data from the measures that all participants completed, excluding those measures that had been used to define the groups (eg, NRS of pelvic pain symptoms). To address aim 2 (stratification of women with CPP into meaningful subgroups), we then focussed only on those with CPP. Our stage 2 analyses took a data-driven approach to identify subgroups within those with CPP. For this stage, we used data from the physiological assessments and questionnaire measures relevant to underlying pain mechanisms (eg, painDETECT, sleep, psychological variables) but again did not include those that were used to define the groups or that would have been expected to align with specific diagnostic groups (eg, measures related to bladder symptoms). Finally, in stage 3, our analyses aimed to better understand the clinical characteristics of the identified subgroups. For these analyses, we therefore did use our fuller set of clinical data including measures of pain intensity and diagnostic groupings.

#### 2.3.1. Stage I—group-level comparisons

A between-group analysis was run using independent t-tests with Bonferroni correction (*significance at 0.005 level*) to explore any significant effects between the CPP and control groups. The variables included at this stage were questionnaire measures of Sleep and Fatigue scores, mental wellbeing (anxiety and depression scores), personality and pain catastrophising (PCS) as well as physiological and biological measures (average R-R intervals, heart rate variability [HRV], cortisol profiles, CPM response, QST profiles). All the standardised questionnaire data were scored as per the published algorithms for each questionnaire (Table 1). At this stage, the painDETECT and Widespreadness assessments were not included as only CPP participants were asked to complete them.

All physiological data were processed and analysed as per recommended guidelines in the literature. For QST data, raw values were z-transformed as per published literature using age- and sex-matched reference data for the foot (control site)^45^ and trunk (test site),^43^ and z-values are as such that positive numbers show a gain of function and negative a loss of function.

The CPM effect was quantified using both the absolute difference in pressure pain threshold (PPT2_average_ − PPT1_average_) and the percentage change [(PPT2_average_ − PPT1_average_)/PPT1_average_] × 100.^28^ The absolute difference was used to determine the presence of a “true” CPM effect. R-R intervals and HRV were calculated from the ECG recordings for the first 5 minutes pre and post the CPM paradigm using LabChart software, with both baseline and change in R-R interval calculated. The saliva samples for the cortisol profiles were analysed to extract cortisol levels (nmol/L) for pre and post the CPM paradigm, as well as for the 24-hour profile. For the latter, the area under the curve was calculated between the 5 time-points.

#### 2.3.2. Stage II—data-driven chronic pelvic pain stratification

A latent profile analysis (LPA) was used to stratify CPP participants based on a selected set of the measures used in the study. Latent profile analysis is a statistical model used to identify profiles within a heterogeneous population based on a set of continuous variables. Therefore, the selected variables were composed of a set of continuous measures that were either found to be significantly different to the control group or were considered potentially mechanistically important due to evidence from other published literature. As we were aiming to identify mechanistically relevant subgroups, we specifically did not include clinical variables such as pain intensity or diagnostic category, nor did we include quality of life (QoL) measures (Fig. 1).

The data set was preprocessed, and missing values were imputed using mean imputation. The set of selected variables were then standardized into z-scores (mean of 0 and standard deviation of 1). The LPA analysis was conducted using the Gaussian Mixture Model approach with 1 to 5 profiles fitted to the data to calculate the Bayesian Information Criterion (BIC) and Akaike Information Criterion (AIC) to determine the optimal number of clusters. The optimal number of clusters was selected by comparing BIC and AIC across values across different numbers of profiles, and the model with the lowest BIC was selected for further analysis. In addition, we adopted a minimum profile size criterion, ensuring that each profile contained at least 5% of the total sample to reduce the risk of spurious classes.^38^

In addition to LPA, we also conducted an exploratory K-means clustering as a sensitivity analysis to evaluate the robustness of the identified profiles using the same variables as LPA. Because K-means requires the number of clusters to be specified in advance, we set k to align with the number of clusters identified in LPA results. K-means clustering also requires complete data across all variables, and therefore, this analysis was restricted to the subset of participants with no missing values (n = 21). The resulting clusters were compared with the LPA profiles using agreement indices Adjusted Rand Index, Normalized Mutual Information.

#### 2.3.3. Stage III—exploration of the identified subgroups

The final stage of analysis aimed to explore clinically relevant differences between the identified subgroups. Analysis of variances (ANOVAs), post hoc Tukey HSD (Honestly Significant Difference) tests, and Kruskal–Wallis H tests were used for comparisons between the profiles regarding diagnostic group (EAP, EABP, BPS, PP), pelvic pain symptoms, bladder sensitivity, comorbidities, QoL, and pain interference in work, daily activities, sleep, exercising, and social activities.

## 3. Results

### 3.1. Stage I—group-level comparisons

**Figure 2.**
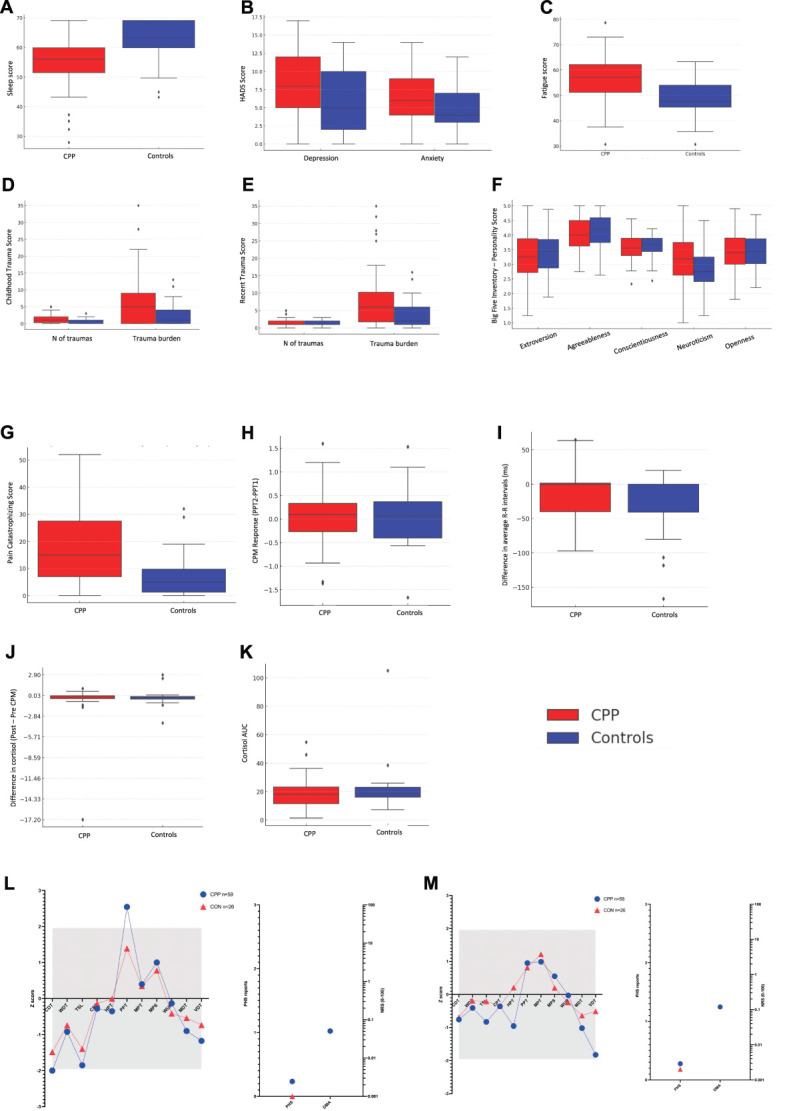
Analysis stage I. Group comparisons between women with chronic pelvic pain (CPP; red) and controls (blue) across domains. (A) Sleep quality (ASCQ-Me v2 Sleep Impact Short Form); (B) Anxiety and depression symptoms (Hospital Anxiety and Depression Scale); (C) Fatigue levels (Neuro-QOL v1 Fatigue); (D and E) Childhood and recent trauma exposure, reported as number of events and burden scores; (F) Big 5 personality traits (Extraversion, Agreeableness, Conscientiousness, Neuroticism, and Openness); (G) Pain catastrophizing (Pain Catastrophizing Scale total score); (H) Conditioned pain modulation (CPM) response using pressure pain thresholds (PPT); (I) Autonomic reactivity as measured by changes in heart rate variability (average R-R intervals); (J) Salivary cortisol response to pain stimulus (pre–post CPM difference); (K) Salivary cortisol levels 24 hours profile(area under the curve; AUC); (L and M) Z-score profiles of somatosensory function from quantitative sensory testing (QST) on the lower abdomen (L) and foot (M), including thermal, mechanical, and pain sensitivity. Boxplots depict group medians, interquartile ranges, and outliers.

Comparing those with CPP with controls, we identified significant differences (*P* < 0.005) between the groups for all questionnaire measures except the Big 5 Inventory for personality. Specifically, those with CPP reported higher levels of fatigue (t = 5.16, *P* < 0.001, d = 0.95, CI [0.57-1.32]); poorer sleep (t = −4.21, *P* < 0.001, d = −0.78, CI [−1.15 to 0.40]); higher levels of: anxiety (t = 2.94, *P* = 0.004, d = 0.54, CI [0.17-0.91]), depression (t = 2.88, *P* = 0.005, d = 0.53, CI [0.16-0.90]), and pain catastrophising (t = 6.39, *P* < 0.001, d = 0.91, CI [0.56-1.27]); and more gastrointestinal symptoms apart from reflux and diarrhoea (*P* > 0.005): abdominal pain (t = 3.32, *P* < 0.001, d = 0.51, CI [0.14, 0.86]), indigestion (t = 4.42, *P* < 0.001, d = 0.65, CI [0.29, 1.01]), constipation (t = 3.82, *P* < 0.001, d = 0.58, CI [0.22, 0.94]), and GSRS Total (t = 4.03, *P* < 0.001, d = 0.62, CI [0.25, 0.98]) (Fig. 2 and Table 2).

**Table 2:** Stage I statistical analysis results from independent *t*-tests comparing between participants with chronic pelvic pain and controls.

Assessment	CPP	Controls	*t*	df	*P*	Cohen d	95% confidence interval	
							Lower	Upper
Fatigue score	56.98	48.45	5.16	140	<0.001	1.01	0.569	1.323
HADS depression	8.20	5.96	2.88	128	0.01	0.54	0.162	0.896
HADS anxiety	6.72	5.16	2.94	128	0.00	0.61	0.173	0.907
Sleep	55.37	61.93	−4.21	138	<0.001	−0.71	−1.147	−0.401
Personality extroversion	3.27	3.41	−1.02	140	0.31	−0.20	−0.548	0.174
Personality agreeableness	4.02	4.13	−1.13	140	0.26	−0.14	−0.568	0.154
Personality consientiousness	3.57	3.61	−0.66	140	0.51	−0.07	−0.482	0.239
Personality neuroticism	3.17	2.84	2.16	140	0.03	0.28	0.033	0.759
Personality openness	3.45	3.47	−0.15	140	0.88	0.03	−0.389	0.332
PCS total	17.66	7.12	5.30	151	<0.001	0.76	0.559	1.265
GSRS Reflux	1.67	1.32	1.69	141	0.09	0.25	−0.052	0.658
GSRS abdominal	2.15	1.61	2.76	137	0.01	0.47	0.14	0.86
GSRS indigestion	2.34	1.66	3.63	141	<0.001	0.58	0.291	1.013
GSRS diarrhoea	1.75	1.39	2.05	140	0.04	0.39	0.012	0.724
GSRS constipation	2.19	1.48	3.22	141	0.00	0.53	0.22	0.938
GRSR total score	1.99	1.49	3.39	136	<0.001	0.59	0.251	0.978
Cortisol AUC	18.65	21.11	−0.92	95	0.36	−0.20	−0.627	0.229
Cortisol (mean difference post − pre CPM)	−0.51	−0.24	−0.57	79	0.57	−0.14	−0.602	0.332
R-R intervals (mean difference post − pre CPM/ms)	−12.67	−31.3	1.82	74	0.07	0.44	−0.041	0.926
CPM response	0.07	0.07	0.01	82	0.99	0.63	−0.466	0.47

Values represent group means for each assessment, followed by results from independent samples *t*-tests: *t*-value, degrees of freedom (df), associated *P*-value, Cohen d (effect size), and the 95% confidence interval for the effect size (lower and upper bounds).

AUC, area under the curve; CPM, conditioned pain modulation; CPP, chronic pelvic pain; GSRS, gastrointestinal symptom rating scale; HADS, hospital anxiety and depression scale; PCS, pain catastrophizing scale.

By contrast, we found only limited differences between the groups for the physiological measures assessed. Four components of the QST paradigm were significantly different, with the CPP group exhibiting loss of function for thermal sensory limen on both the abdomen (t = −22.8, *P* = 0.032) and foot (and t = −3.5, *P* = 0.004), and vibration detection on the foot (t = −3.0, *P* = 0.017) and gain of function for pressure pain threshold on the abdomen (t = −3.0, *P* = 0.012).^9^ There were no significant differences in measures of autonomic nervous system activity, CPM, or cortisol profiles (*P* > 0.01) (Fig. 2 and Table 2).

### 3.2. Stage II—data-driven chronic pelvic pain stratification

**Figure 3.**
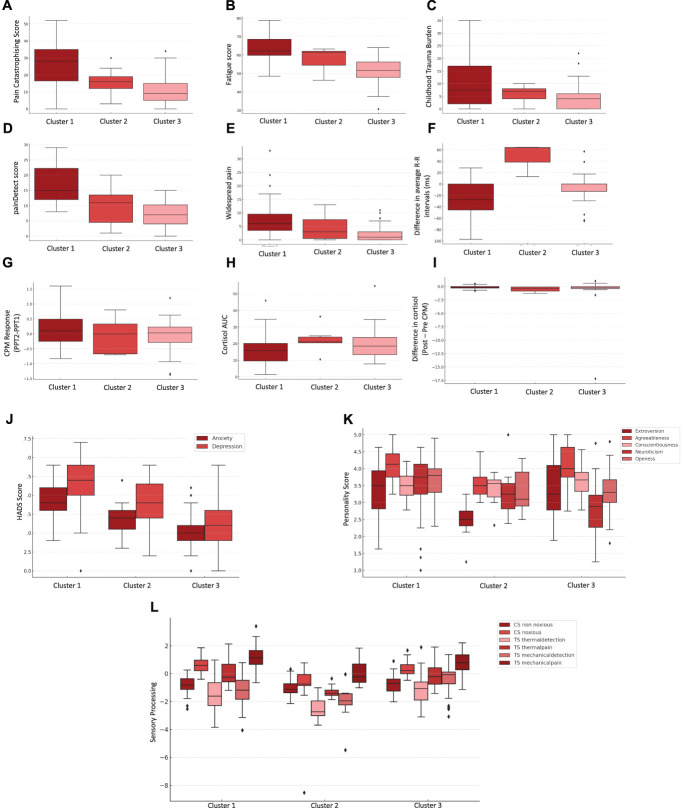
Analysis stage II. Differences across 3 latent profile analysis (LPA) clusters (Cluster 1 = dark red, Cluster 2 = medium red, Cluster 3 = light red) across domains. (A) Pain catastrophizing (Pain Catastrophizing Scale); (B) Fatigue (Neuro-QOL v1 Fatigue t-score); (C) Childhood trauma burden; (D) Pain nature (painDETECT score); (E) Pain widespreadness (number of body areas with persistent pain); (F) Autonomic function measured by heart rate variability (difference in average R-R intervals pre- and post-CPM); (G) Conditioned pain modulation (CPM) response using pressure pain thresholds (PPT); (H) Salivary cortisol levels 24 hours profile (area under the curve [AUC]); (I) Salivary cortisol response to pain stimulus (pre–post CPM difference); (J) Anxiety and depression (HADS subscales); (K) Personality dimensions (Big 5 Inventory: Extraversion, Agreeableness, Conscientiousness, Neuroticism, Openness); (L) z-scores from QST domains: CS non-noxious, CS noxious, TS thermal detection, TS thermal pain, TS mechanical detection, and TS mechanical pain. Boxplots show the distribution of scores per cluster with median, interquartile range, and outliers.

Based on the results of the LPA analysis, the 3-cluster model was selected as the best solution because it had the lowest values for both the BIC (BIC = 6367.85) and the AIC (AIC = 3956.61) compared with models with fewer or more profiles. These values indicate that the model achieves the best balance between goodness-of-fit and model simplicity. The 3 identified clusters were characterized by distinct patterns of means across variables included in the analysis (Fig. 3 and Table 3) and included: 43 participants in cluster 1, 11 participants in cluster 2, and 54 participants in cluster 3.

**Table 3:** Means of all study variables for each latent profile analysis cluster and results of 1-way analysis of variances comparing the 3 clusters.

Variable	Cluster 1	Cluster 2	Cluster 3	ANOVA	Effect size	95% confidence interval	
	Mean	Mean	Mean	*P*	Cohen D	Lower	Upper
Fatigue score	63.88	58.02	51.36	**0.001**	**0.403**	**0.247**	**0.514**
HADS depression	11.21	9	6.07	**0.001**	**0.31**	**0.144**	**0.439**
HADS anxiety	9.21	6.82	5.09	**0.001**	**0.4**	**0.23**	**0.52**
Sleep	50.46	56.49	58.75	**0.001**	**0.187**	**0.057**	**0.31**
Personality extroversion	3.37	2.48	3.36	**0.002**	**0.121**	**0.019**	**0.236**
Personality agreeableness	4.08	3.56	4.08	0.011	0.089	0.005	0.196
Personality conscientiousness	3.52	3.4	3.64	0.156	0.038	0	0.122
Personality neuroticism	3.56	3.31	2.84	**0.001**	**0.167**	**0.045**	**0.288**
Personality openness	3.66	3.34	3.31	0.024	0.074	0	0.177
PCS total	27	15.91	10.53	**0.001**	**0.354**	**0.201**	**0.469**
GSRS reflux	1.81	1.77	1.55	0.634	0.01	0	0.064
GSRS abdominal	2.76	2.27	1.69	**0.001**	**0.167**	**0.042**	**0.292**
GSRS indigestion	2.68	2.66	2.02	0.02	0.079	0.001	0.185
GSRS diarrhoea	1.85	2.54	1.49	**0.008**	**0.098**	**0.008**	**0.209**
GSRS constipation	2.65	2.18	1.85	0.025	0.075	0	0.179
GRSR total score	2.3	2.29	1.71	**0.007**	**0.103**	**0.009**	**0.219**
Cortisol AUC	16.09	22.42	20.32	0.139	0.061	0	0.181
Difference average RR (ms)	−26.33	47	−5.6	**0.001**	**0.268**	**0.063**	**0.43**
Dysmenorrhoea	7.17	5.75	5.62	0.129	0.091	0	0.248
Dyspareunia during intercourse	6.26	4.5	3.66	**0.003**	**0.167**	**0.024**	**0.311**
Dyspareunia postintercourse	5.37	3.67	2.26	**0.001**	**0.225**	**0.059**	**0.371**
Pelvic pain	6.59	5.5	4.68	**0.003**	**0.145**	**0.021**	**0.278**
Widespread pain	7.64	4.36	2.32	**0.001**	**0.199**	**0.067**	**0.322**
PainDETECT	16.4	9.73	7.26	**0.001**	**0.394**	**0.239**	**0.505**
CPM response (PPT2 - PPT1)	0.27	−0.05	−0.11	0.071	0.09	0	0.23
QST CDT (test site)	−1.9677	−2.168	−1.9971	0.955	0.002	0	0.02
QST WDT (test site)	−0.5962	−0.832	−1.2343	0.396	0.033	0	0.14
TSL (test site)	−1.7888	−1.96	−1.8893	0.932	0.003	0	0.034
CPT (test site)	−0.1908	−0.204	−0.3729	0.782	0.009	0	0.077
HPT (test site)	−0.2196	−0.592	−0.4282	0.652	0.015	0	0.099
MDT (test site)	−1.1015	−0.824	−0.7236	0.365	0.035	0	0.145
MPT (test site)	0.3723	−0.29	0.5454	0.415	0.031	0	0.137
MPS (test site)	0.9292	1.262	1.0211	0.922	0.003	0	0.039
DMA (test site)	0.1485	0.108	0.1393	0.896	0.004	0	0.049
WUR (test site)	0.0142	−0.285	−0.2475	0.786	0.009	0	0.077
VDT (test site)	−1.3904	−2.815	−0.7361	0.104	0.079	0	0.216
PPT (test site)	3.5723	2.2575	1.6346	**0.002**	**0.204**	**0.034**	**0.36**
CDT (control site)	−0.6964	−0.964	−0.7815	0.915	0.003	0	0.043
WDT (control site)	−0.4608	−0.28	−0.3833	0.961	0.001	0	0.016
CPT (control site)	−0.2304	−0.952	−0.3774	0.279	0.045	0	0.164
HPT (control site)	−0.0758	−0.294	−1.9237	0.542	0.022	0	0.118
MDT (control site)	−1.1446	−1.2	−0.8667	0.506	0.024	0	0.124
MPT (control site)	1.23	0.87	0.78	0.357	0.037	0	0.149
MPS (control site)	0.47	0.95	0.56	0.778	0.009	0	0.079
DMA (control site)	0.14	0.13	0.27	0.722	0.012	0	0.089
WUR (control site)	−0.0062	−0.072	−0.0419	0.991	0	0	0
VDT (control site)	−2.0285	−1.2275	−1.717	0.785	0.009	0	0.079
PPT (control site)	1.2	0.74	0.75	0.415	0.032	0	0.141
Heart rate variability (HRV) (ms)	3.82	3.58	4.05	0.342	0.044	0	0.169
Average RR (ms) at baseline	882.66	789.3	929.27	0.139	0.079	0	0.224
Cortisol (nmol) timepoint 1	6.69	9.42	8.29	0.297	0.038	0	0.145
Cortisol (nmol) timepoint 2	8.09	12.07	9.79	0.33	0.036	0	0.141
Cortisol (nmol) timepoint 3	3.02	3.68	3.21	0.867	0.005	0	0.053
Cortisol (nmol) timepoint 4	2.16	1.48	2.35	0.768	0.009	0	0.075
Cortisol (nmol) timepoint 5	0.89	0.95	2.12	0.069	0.086	0	0.222
Cortisol morning rise (timepoint 2 − timepoint 1)	1.17	2.65	1.48	0.89	0.004	0	0.048
SF36 physical functioning	68.81	91.82	92.22	**0.001**	**0.278**	**0.132**	**0.398**
SF36 physical health limitations	27.44	54.55	63.24	**0.001**	**0.146**	**0.034**	**0.263**
SF36 emotional limitations	23.58	27.27	45.1	0.045	0.06	0	0.156
SF36 energy fatigue	33.45	45.45	55.39	**0.001**	**0.315**	**0.165**	**0.433**
SF36 emotional wellbeing	47.41	53.33	63.33	**0.001**	**0.183**	**0.058**	**0.303**
SF36 social functioning	47.62	70.46	78.28	**0.001**	**0.237**	**0.098**	**0.358**
SF36 pain	37.44	68.18	65.44	**0.001**	**0.219**	**0.084**	**0.341**
SF36 general health	33.43	41	57.25	**0.001**	**0.198**	**0.069**	**0.319**
Pain interference with daily activities	3	2.25	1.65	**0.001**	**0.328**	**0.147**	**0.463**
Pain interference with sleep	3.22	2.63	1.85	**0.001**	**0.29**	**0.118**	**0.426**
Pain interference with sexual intercourse	3.89	3.33	2.31	**0.001**	**0.254**	**0.09**	**0.391**
Pain interference with exercise sport	3.35	2.75	1.87	**0.001**	**0.233**	**0.057**	**0.384**
Pain interference with social activities	2.76	2.43	1.63	**0.001**	**0.312**	**0.132**	**0.449**
Pain interference with work/school	3.11	1.86	1.62	**0.001**	**0.211**	**0.054**	**0.352**
Bladder pain intensity at first sensation (_/10)	3.02	3.67	0.77	**0.001**	**0.84**	**−3.413**	**2.126**
Bladder pain intensity at first urge (_/10)	3.6	5	1.74	**0.005**	**0.525**	**−4.532**	**1.722**
Possible interpretation of type of pain	**Nociplastic**	**?**	**Nociceptive**				

Significant results (*P* < 0.05) are shown in bold.

ANOVA, analysis of variance; AUC, area under the curve; CPM, conditioned pain modulation; GSRS, gastrointestinal symptom rating scale; HADS, hospital anxiety and depression scale; PCS, pain catastrophizing scale; QST, quantitative sensory testing; SF-36, 36-Item Short Form Health Survey.

An ANOVA revealed significant differences between clusters for a number of variables across our domains. Specifically, we found significant differences in questionnaire measures of: PCS (F [2, 105] = 20.57, *P* < 0.0001), fatigue scores (F [2, 105] = 15.92, *P* < 0.0001), depression (F [2, 105] = 14.39, *P* < 0.0001), anxiety levels (F [2, 105] = 16.21, *P* < 0.0001), and the neuroticism domain of the personality scale (F [2, 105] = 10.90, *P* < 0.0001). Similarly, significant differences were observed in pain-relevant measures of the painDETECT questionnaire (F [2, 105] = 11.55, *P* < 0.0001) and Widespreadness (F [2, 105] = 9.88, *P* < 0.001).

However, statistical analysis of the physiological measures revealed significant effects only for certain QST measures: thermal pain on the test site (F [2, 105] = 9.62, *P* < 0.001), thermal detection on the test site (F [2, 105] = 6.38, *P* < 0.01), and noxious stimuli on the control site (F [2, 105] = 7.47, *P* < 0.01). No significant measures were observed in any of the other physiological measures.

Post hoc Tukey HSD tests were conducted for the observed significant effects (Fig. 3) to determine which specific clusters differed from each other. In summary, cluster 1 scored significantly higher compared with cluster 3 on the painDetect scale as well as measures of widespreadness, pain catastrophising, fatigue, anxiety and depression, and the neuroticism domain of the personality scale. Moreover, cluster 1 had significantly higher scores than cluster 3 on the QST measures of thermal pain sensitivity (test site), thermal detection (test site), and noxious stimuli detection (control site). The only significant difference between cluster 1 and cluster 2 was a higher score of pain catastrophising in cluster 1. No significant differences were observed between clusters 2 and 3 across any of the variables.

The K-means model was run with 3 clusters to align with the solution identified by LPA, using the same 22 standardized variables. Because K-means requires complete data across all variables, this analysis was limited to participants with no missing values (n = 21). The resulting clusters showed partial but not strong agreement with the LPA profiles, with an Adjusted Rand Index (ARI) of 0.22 and a Normalized Mutual Information (NMI) of 0.37. Despite these modest agreement metrics, the overlap table demonstrated a reasonable correspondence between the 2 methods: participants in LPA cluster 1 were mostly classified into K-means cluster 3, while those in LPA cluster 3 were split between clusters 1 and 2 (see Supplementary Table S1, http://links.lww.com/PAIN/C416).

### 3.3. Stage III—exploration of the identified subgroups

#### 3.3.1. Diagnostic group

**Figure 4.**
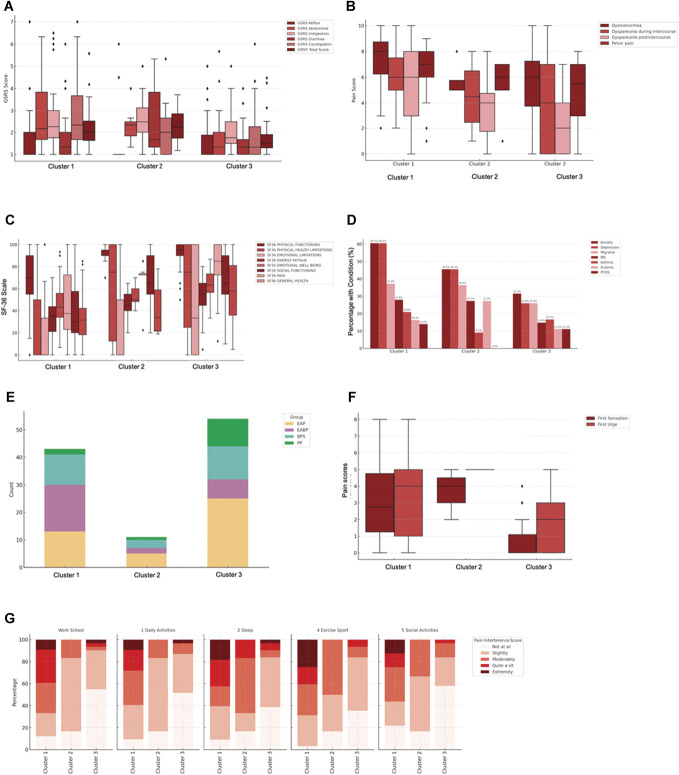
Analysis stage III. Clusters 1, 2, and 3 as defined by the LPA analysis across clinically relevant measures. Cluster groups are colored in shades of red, from darkest (Cluster 1) to lightest (Cluster 3). (A) Gastrointestinal Symptom Rating Scale (GSRS) scores by cluster for the assessed GSRS domains; (B) Scores for pain domains: dysmenorrhea, dyspareunia during intercourse, dyspareunia postintercourse, and pelvic pain during gynaecological examination; (C) SF-36 quality of life domain scores: physical functioning, physical health limitations, emotional limitations, energy/fatigue, emotional wellbeing, social functioning, pain, and general health; (D) Percentage of participants in each cluster reporting clinical comorbidities (anxiety, depression, IBS, migraine, asthma, eczema, PCOS); (E) Distribution of clinical diagnostic categories across clusters; (F) Pain ratings at first bladder sensation and first urge (visceral sensitivity); (G) Stacked bar chart showing percentage distribution of pain interference ratings (Not at all—Extremely) across 6 domains: work/school, daily activities, sleep, exercise/sports, and social activities. All boxplots show medians, interquartile ranges, and outliers. BPS, bladder pain syndrome; EABP, endometriosis-associated bladder pain; EAP, endometriosis-associated pain; PP, primary pain; SF-36, 36-item short form health survey.

Descriptive exploration of the clusters regarding the clinical diagnostic group of the patients (EAP, EABP, BPS, PP) showed that all diagnostic groups were represented in each cluster (cluster 1: EAP = 30.2%, EABP = 39.5%, BPS = 25.6%, PP = 4.7%; cluster 2: EAP = 45.5%, EABP = 18.2%, BPS = 27.3%, PP = 9.1%; cluster 3: EAP = 46.3%, EABP = 13%, BPS = 22.2%, PP = 18.5%) (Fig. 4).

Similarly, participants of each diagnostic group were spread between the 3 clusters (EAP: cluster I: 30%, cluster II: 11.6%, cluster III: 58.1%; EABP: cluster I: 64.5%, cluster II: 7.7%, cluster III: 26.9%; BPS: cluster I: 42.3%, cluster II: 11.5%, cluster III: 46.2%; PP: cluster I: 15.4%, cluster II: 7.7%, cluster III: 76.9%).

#### 3.3.2. Pelvic pain symptoms

The results from 1-way ANOVAs revealed significant effects for dyspareunia during intercourse (F [2,67] = 6.48, *P* = 0.003), dyspareunia postintercourse (F [2,67] = 6.50, *P* < 0.001), and noncyclical pelvic pain (F [2,78] = 6.48, *P* = 0.003); but not for dysmenorrhea (F [2,103] = 2.15, *P* = 0.129) (Fig. 4).

Post hoc comparisons using the Tukey HSD test indicated that cluster 1 had significantly higher pain intensity levels than cluster III for dyspareunia during intercourse (*P* = 0.002, CI [0.87, 4.34]), dyspareunia postintercourse (*P* < 0.001, CI [1.40, 4.83]) and noncyclical pelvic pain (*P* = 0.002, CI [0.64, 3.20]).

#### 3.3.3. Gastrointestinal symptoms

A 1-way ANOVA showed significant differences between the profiles for the abdominal pain (F [2,93] = 9.11, *P* < 0.001) and diarrhoea (F [2,96] = 5.09, *P* < 0.008) subscale of the GSRS questionnaire as well as the total score (F [2,92] = 5.19, *P* = 0.007), but not for any of the other subscales (reflux, indigestion, and constipation) (*P* > 0.008) (Fig. 4).

Post hoc comparisons using the Tukey test revealed that the cluster I scored significantly higher on the abdominal pain (*P* < 0.001, CI [−1.67, −0.47]) and total GSRS score (*P* = 0.011, CI [0.12, 1.06]) compared with cluster III. While in the diarrhoea subscale, cluster II scored significantly higher than cluster III (*P* = 0.007, CI [0.24, 1.87]).

#### 3.3.4. Bladder sensitivity testing

A 1-way ANOVA test was conducted to explore effects between the clusters, revealing significant effects in pain intensity at first sensation (F [2, 43] = 9.20, *P* < 0.001) and first urge (F [2, 42] = 5.92, *P* = 0.005). Post hoc tests showed that cluster I and cluster II had significantly higher pain intensity scores than cluster III for first sensation (vs cluster I *P* < 0.001 CI: −3.67 to 0.85, vs cluster II *P* = 0.045 CI: −5.75 to 0.05) and first urge (vs cluster I *P* = 0.018 CI: −3.46 to 0.25, vs cluster II *P* = 0.045 CI: −6.47 to 0.05).

#### 3.3.5. Physiological measures (cortisol and heart rate variability)

A 1-way ANOVA revealed no significant differences between the 3 clusters for any of the 5 cortisol measurement timepoints, nor for the morning rise (timepoint 2 − timepoint 1) (all *P* > 0.05) (Table 4). Similarly, no significant differences were observed in baseline HRV between the clusters (*P* > 0.05) (Table 4).

**Table 4:** Stages II and III statistical analysis results of 1-way analysis of variances comparing across the 3 clusters on the latent profile analysis.

Assessment	F	df between	df within	*P*	Eta squared (η^2^)	95% CI of η^2^
Dysmenorrhea	2.151	2	43	0.129	0.024	−0.022, 0.117
Dyspareunia during intercourse	6.497	2	65	0.003	0.075	−0.003, 0.167
Dyspareunia postintercourse	9.446	2	65	<0.001	0.11	0.015, 0.211
Pelvic pain	6.467	2	76	0.003	0.065	−0.002, 0.147
Pelvic gynae examination	3.764	2	73	0.028	0.035	−0.013, 0.108
PCS total	27.387	2	100	<0.001	0.204	0.101, 0.295
SF36 physical functioning	19.436	2	101	<0.001	0.151	0.060, 0.238
SF36 physical health limitations	8.531	2	100	<0.001	0.068	0.007, 0.141
SF36 emotional limitations	3.192	2	100	0.045	0.021	−0.010, 0.074
SF36 energy fatigue	23.194	2	101	<0.001	0.176	0.079, 0.265
SF36 emotional wellbeing	11.305	2	101	<0.001	0.09	0.020, 0.168
SF36 social functioning	15.658	2	101	<0.001	0.124	0.041, 0.207
SF36 pain	14.19	2	101	<0.001	0.113	0.034, 0.194
SF36 general health	12.464	2	101	<0.001	0.099	0.025, 0.179
Fatigue score	32.735	2	97	<0.001	0.241	0.130, 0.335
HADS depression	18.413	2	82	<0.001	0.17	0.065, 0.268
HADS anxiety	27.378	2	82	<0.001	0.237	0.117, 0.338
Sleep	10.907	2	95	<0.001	0.092	0.019, 0.172
Widespreadness TRiPP	12.049	2	97	<0.001	0.099	0.024, 0.181
PainDetect	31.795	2	98	<0.001	0.234	0.124, 0.327
Cortisol AUC	2.034	2	63	0.139	0.015	−0.015, 0.083
GSRS Reflux	0.459	2	95	0.634	−0.006	−0.010, 0.022
GSRS abdominal	9.115	2	91	<0.001	0.079	0.010, 0.159
GSRS indigestion	4.074	2	95	0.02	0.03	−0.010, 0.091
GSRS diarrhoea	5.085	2	94	0.008	0.04	−0.007, 0.106
GSRS constipation	3.837	2	95	0.025	0.028	−0.010, 0.087
GRSR total score	5.19	2	90	0.007	0.043	−0.007, 0.111
Cortisol difference pre post CPM	0.459	2	52	0.635	−0.01	−0.019, 0.038
24 h cortisol profile − Morning rise (timepoint 2 − timepoint 1)	0.117	2	62	0.89	0.004	0.00, 0.048
24 h cortisol profile − Timepoint 1	1.238	2	62	0.297	0.038	0.00, 0.145
24 h cortisol profile − Timepoint 2	1.13	2	61	0.33	0.036	0.00, 0.141
24 h cortisol profile − Timepoint 3	0.143	2	61	0.867	0.005	0.00, 0.053
24 h cortisol profile − Timepoint 4	0.265	2	60	0.768	0.009	0.00, 0.075
24 h cortisol profile − Timepoint 5	2.791	2	59	0.069	0.086	0.00, 0.222
Baseline heart rate variability (HRV)	1.098	2	48	0.342	0.044	0.00, 0.169
Difference average RR ms 5 min	8.783	2	48	<0.001	0.132	0.012, 0.252
CPM response PPT2 PPT1	2.769	2	56	0.071	0.029	−0.017, 0.111
Personality extroversion	6.68	2	97	0.002	0.054	−0.001, 0.123
Personality agreeableness	4.715	2	97	0.011	0.036	−0.008, 0.098
Personality conscientiousness	1.892	2	97	0.156	0.009	−0.010, 0.054
Personality neuroticism	9.709	2	97	<0.001	0.08	0.013, 0.157
Personality openness	3.882	2	97	0.024	0.028	−0.010, 0.086
Time to first urge (min)	0.57	2	42	0.57	−0.01	−0.023, 0.052
Pain intensity at first sensation (/10)	9.2	2	43	<0.001	0.151	0.016, 0.278
Volume voided (mL)	2.934	2	44	0.064	0.04	−0.022, 0.139

Each row presents the F-statistic, degrees of freedom between and within groups, associated *P*-value, effect size (Eta Squared, η^2^), and the 95% confidence interval of η^2^ for each variable.

AUC, area under the curve; CPM, conditioned pain modulation; GSRS, gastrointestinal symptom rating scale; HADS, hospital anxiety and depression scale.

#### 3.3.6. Comorbidities

Across all 3 clusters, the most common comorbidities reported by more than 10% of all the CPP participants were anxiety, depression, migraine, IBS, asthma, eczema, and polycystic ovary syndrome (PCOS). Chi-square test of independence used to compare frequencies of these comorbidities between clusters revealed a significant effect for the depression diagnosis (χ^2^ [2] = 11.82, *P* = 0.003, Cramer V [effect size] = 0.331). No other significant differences were identified.

#### 3.3.7. Quality of life (36-item short form)

One-way ANOVAs of the 36-item short form (SF-36) subscales revealed significant effects between the clusters for Physical Functioning (F [2,103] = 19.43, *P* < 0.001), Physical Health Limitations (F [2,102] = 8.53, *P* < 0.001), Energy/Fatigue (F [2,103] = 23.19, *P* < 0.001), Emotional Wellbeing (F [2,103] = 11.31, *P* < 0.001), Social Functioning (F [2,103] = 15.66, *P* < 0.001), Pain (F [2,103] = 14.19, *P* < 0.001), and General Health (F [2,103] = 12.46, *P* < 0.001) but not for Emotional Limitations (F [2,102] = 3.20, *P* = 0.045).

Post hoc comparisons using the Tukey HSD test indicated that cluster 1 had significantly lower QoL for Physical Functioning (*P* < 0.001, CI [−32.70, −14.13]), Physical Health Limitations (*P* < 0.001, CI [−56.60, −15.00]), Energy/Fatigue (*P* < 0.001, CI [−32.70, −14.13]), Emotional Wellbeing (*P* < 0.001, CI [−29.60, −14.28]), Social Functioning (*P* < 0.001, CI [−43.81, −17.52]), Pain (*P* < 0.001, CI [−41.29, −14.71]), and General Health (*P* < 0.001, CI [−35.31, −12.35]). No significant differences were observed between clusters 2 and 3.

#### 3.3.8. Pain interference

Kruskal–Wallis H tests were used to compare pain interference between clusters. Significant differences were observed across clusters in interference related to work/school activities, H(2) = 19.48, *P* < 0.001; daily activities, H(2) = 16.29, *P* < 0.001; sleep, H(2) = 10.99, *P* = 0.0004; sexual intercourse, H(2) = 13.55, *P* = 0.001; and exercise or sport, H(2) = 18.86, *P* < 0.001. Post hoc pairwise Mann–Whitney U tests with Bonferroni correction (*P* < 0.01) indicated that participants in cluster 1 reported significantly greater pain interference in work/school (U = 192.5, *P* < 0.001, r ≈ 0.51) and daily activities (U = 210.0, *P* < 0.001, r ≈ 0.53) compared with cluster 3.

## 4. Discussion

As expected, our study demonstrated perturbations in pain-relevant systems between women with CPP and pelvic pain-free women. These differences were more pronounced for questionnaire measures than physiological tests. Moreover, we demonstrated that it is possible to use these measures to identify clusters within our CPP population, which seem to represent different clinical phenotypes that are not driven by a clinical diagnosis of endometriosis or IC/BPS.

Although all the questionnaire measures were able to identify significant differences between women with CPP and controls, it was surprising that, from the physiological measures only, specific QST components differed between the 2 groups.^9^ Our results are in contrast to the existing literature for other chronic pain conditions such as IBS, low back pain, and fibromyalgia in which physiological measures such as CPM, cortisol, and ECG do differ significantly when compared with a healthy population.^1,2,19,20,26,39,42,46^ However, it should be noted that in our cohort, there is wide variability across all physiological measures assessed, especially in the women with CPP, potentially explaining the lack of significant difference at a group level. One explanation for this may be the influence of hormonal state on our chosen physiological measures.^10^ Although hormonal variation can also affect some of the measures we assessed with questionnaires (eg, mood, fatigue), most of the tools we chose for these assessments were those that assessed trait rather than state. We specifically chose not to control for endogenous hormonal state or exogenous hormone use, as we aimed to identify a stratification method that was stable across time and useful in clinical settings.

Interestingly, the LPA analysis results reflected this heterogeneity within the CPP group by classifying participants into 3 clusters. However, similar to stage 1 of the analysis, physiological measures of heart rate, cortisol levels, and CPM do not seem to be affecting the identified clusters. Previous work by the MAPP consortium has shown that structural and functional brain differences are associated with distinct symptom subgroups in chronic pelvic pain,^29,50^ suggesting that neuroimaging could provide valuable mechanistic insights into the profiles identified in our study. The original design of TRiPP had aimed to collect fMRI data with the specific aim of exploring potential central nervous system markers of any identified subgroups; however, unfortunately restrictions during and immediately after the COVID-19 pandemic limited the data we were able to collect and thus means we cannot address this question within TRiPP itself. Given the findings presented here and from MAPP previously, we believe this to be an important area for future investigation.

However, the questionnaire measures, including assessments of pain characteristics such as painDETECT and widespreadness, seem to be driving the stratification. Considering clinical translatability, this is an important finding, as the use of questionnaires plus potentially a simplified sensory test would be easier (and cheaper) to implement in a clinical setting than a battery of more complex, time-consuming tests that require expensive equipment/specialist training or lead to a delay in receiving results (eg, cortisol analysis).

We specifically chose not to include measures of symptoms or diagnoses within the data at stage II so that these factors did not influence clustering. Similarly, we excluded measures that might align more with one diagnostic group than another, such as bladder sensitivity and sleep (in case this was disproportionately affected by nocturia). In line with our hypothesis that factors other than the peripheral pathology are of importance in determining subphenotypes, we found our 4 diagnostic groups spread across the 3 clusters. Interestingly, the clusters we have identified align with those found in other studies. Exploring the QoL and pain interference data, our analysis suggests that cluster 1 comprises most of those with high-impact pain. Although it should be noted that high-impact chronic pain was originally defined as persistent pain with “substantial restriction of participation in work, social, and self-care activities for 6 months or more,”^13,31^ more recently a timeframe of 3 months has been used in line with our assessments.^30,44^ The MAPP network recently demonstrated that high-impact pain in a cohort of patients with urological chronic pelvic pain syndrome (64% female) was associated with both widespread pain and pain in response to consuming a standardised volume of water, as well as pelvic floor tenderness (not assessed in our study).^49^ Our findings suggest that these factors are important in those with chronic pelvic pain more broadly not just urological pelvic pain.

Our clusters also have similarities with nociplastic pain (cluster 1) and nociceptive pain (cluster 3). It is important to note that there currently are no diagnostic criteria published for nociplastic pain in visceral pain conditions and some of the criteria used for musculoskeletal pain are perhaps less applicable (eg, most visceral pain is regional in distribution rather than discretely localised).^32^ However, features such as fatigue, widespread pain, psychological distress, and increased sensitivity to a noxious stimulus at a distant site would all be consistent with this classification. On the other hand, cluster 3 demonstrated a low painDETECT score, pain localised to the pelvis, low levels of psychological distress, and low pain scores in response to visceral sensations, all of which would point more to a nociceptive phenotype.^18^

The heterogeneity seen within the clusters in physiological measures potentially suggests that multiple different mechanisms could generate each phenotype. This would be consistent with other published literature suggesting, for example, that both top-down and bottom-up processes can lead to nociplastic pain.^18^ However, recent work by the MAPP network has clearly illustrated the relationship between a widespread pain phenotype in urological chronic pelvic pain and the response to a variety of treatments.^17^ Surgical procedures (including ablation/excision of endometriosis and hysterectomy) that are commonly used in the management of CPP in women are associated with complications and significant financial cost, but are frequently unsuccessful in improving pain.^24^ We therefore believe that there is an urgent need to explore whether this widespread pain phenotype predicts treatment response in a broader population of patients with CPP including those with endometriosis. One study exploring hysterectomy as a treatment for CPP would support this strategy.^5^ To date, there is very limited research on the role of a multidisciplinary pain management approach in CPP despite this being the recommended approach for other forms of chronic pain, particularly when nociplastic features are present.

### 4.1. Limitations

Although our study provides valuable insights, it has some limitations. The rate of recruitment of participants for this part of the TRiPP study was severely affected by the COVID-19 pandemic, and as a result, the sample size is less than originally planned. Although this may contribute to the lack of significant differences seen in the physiological assessments, we would have expected a bigger impact on the questionnaire measures which are arguably less sensitive.

Although, the results of the K-means sensitivity analysis provided additional support for the robustness of the latent profiles, the modest agreement metrics likely reflected the reduced sample size available for complete-case clustering. These findings support the appropriateness of LPA for our data set, while also highlighting the value of complementary approaches for sensitivity testing. However, future research should replicate our findings in larger samples and conduct sensitivity analyses using alternative data-driven clustering approaches to further evaluate their validity.

The cross-sectional design of our study also limits the causal inferences that can be drawn. Future research needs to prospectively validate these clusters and to study them longitudinally to understand their stability and trajectories over time. Equally important is the investigation of how these clusters respond to different treatments. Interestingly, work from the MAPP network does suggest their subgroups are stable over time.^33^

Although clusters 1 and 3 seem to relate to other published work, it is harder to understand cluster 2 particularly when this is smaller than the other clusters. The potential role of the autonomic nervous system and HPA axis in differentiating this cluster (Fig. 3) should be explored in a larger sample. Work in urological pelvic pain also suggests that there is a clinical phenotype characterised by dysfunction of the pelvic floor muscles.^22^ This is a clinical finding seen in association with endometriosis and other types of CPPS too,^40^ and thus, future work should consider determining whether this is a distinct phenotype of CPP. It is believed that the recent addition of a standardised tool for clinical assessments to the EPHect tools^35^ will facilitate collaborative work in this area.

## 5. Conclusion

Our study demonstrates differences in pain-relevant systems between women with CPP and pain-free controls, and how these can be used to stratify women with CPP into subgroups. These subgroups seem to be clinically meaningful and align with work in other forms of chronic pain. We believe that our findings support the need for a different more personalised and more nuanced therapeutic strategy, potentially taking a pain-focused rather than a pelvis-focussed approach to those with high-impact pain. Further clinical research is urgently needed in this area given the significant burden of CPP in women.

## Conflict of interest statement

L.D., L.C., E.E., K.K., D.P., K.P., E.T., A.C., J.F.G., P.A.M., C.E.L., L.A.N., Q.A., J.B., K.G., A.S., L.H., M.K., J.M., C.S., A.F.V.: No competing interests. A.H.: Employee of Bayer AG, Germany. A.W.H.: Dr Horne reports receiving grants from the UK Research and Innovation, UK National Institute for Health and Care Research, Scotland's chief scientist's office, Wellbeing of Women, and Roche Diagnostics; consultancy fees from Roche Diagnostics, Gesynta, and Thramex; lecture fees from Gedeon Richter; having a pending patent (UK Patent 2217921·2); serving as President-elect of the World Endometriosis Society, co–editor in chief of Reproduction and Fertility, trustee and medical adviser to Endometriosis UK, and specialty adviser to the Scottish government's Chief Medical Officer for obstetrics and gynaecology. E.M.P.Z.: Esther M. Pogatzki-Zahn received financial support from Grünenthal, Germany, for research activities and advisory and lecture fees from Grünenthal, Germany, MSD/MERCK, Germany, Merz Pharmaceuticals gmbh, and Medtronic, Switzerland. In addition, she receives scientific support from the German Research Foundation (DFG), the Federal Ministry of Education and Research (BMBF), the Federal Joint Committee (G-BA), and the Innovative Medicines Initiative 2 Joint Undertaking under Grant Agreement No 777500. This Joint Undertaking receives support from the European Union's Horizon 2020 research and innovation program and EFPIA. R.D.T.: Dr. Treede reports grants from IMI2 PainCare project of EU, grants from TEVA, Esteve, during the conduct of the study; personal fees from Bayer, Grünenthal, GSK, Sanofi, Merz, and Vertex, outside the submitted work; In addition, Dr. Treede has a patent DE 103 31 250.1-35 with royalties paid to MRC Systems. J.V.: has received research funding from Viatris and consultancy fees from Grünenthal, AstraZeneca, and Merz Pharmaceuticals, outside the submitted work. C.M.B.: Research grants from Bayer Healthcare, MDNA Life Sciences, Roche Diagnostics, European Commission, NIH. His employer has received consultancy fees from Myovant and ObsEva for work outside of this project. F.C.: Consultant and/or investigator for Allergan (AbbVie), Astellas, Bayer, Ipsen, and Recordati. S.A.M.: Advisory board member for AbbVie and Roche; receives research funding from the National Institutes of Health, the US Department of Defence, the J. Willard and Alice S. Marriott Foundation, and AbbVie. None are related to the presented work. K.Z.: Reports grant funding from EU Horizon 2020, NIH US, Wellbeing of Women, Bayer AG, Roche Diagnostics, Evotec-Lab282, and MDNA Life Sciences, outside the submitted work. J.N.: Employee of Merz Therapeutics GmbH and shareholder of Bayer AG, Germany, and shareholder of Eli Lilly. K.V.: Declares research funding from UKRI, NIHR, NIH US, and Bayer AG outside of the submitted work, and honoraria for consultancy and talks and associated travel expenses paid to her institution from Bayer AG, AbbVie, Reckitts, and Eli Lilly.

## Appendix A. Supplemental digital content

Supplemental digital content associated with this article can be found online at http://links.lww.com/PAIN/C416.

## Supplementary Material

SUPPLEMENTARY MATERIAL
