# Supplementary material for: Women with chronic pelvic pain can be stratified using multimodal assessment
Source: Pain. 2025 Nov 17;167(4):786–802. doi: 10.1097/j.pain.0000000000003857 (PMC12994511; doi:10.1097/j.pain.0000000000003857)
Supplement: SUPPLEMENTARY MATERIAL [file jop-167-786-s002.pdf]

	K-means Cluster 1	K-means Cluster 2	K-means Cluster 3	All
**LPA Cluster 1**	1 (2.3%)	2 (4.7%)	5 (11.6%)	43 (100%)
**LPA Cluster 2**	2 (18.2%)	0 (0.0%)	0 (0.0%)	11 (100%)
**LPA Cluster 3**	5 (9.3%)	6 (11.1%)	0 (0.0%)	54 (100%)
**All**	8 (7.4%)	8 (7.4%)	5 (4.6%)	108 (100%)

Supplementary table 1. *Overlap between latent profile analysis (LPA) and K-means clusters (n = 21 complete cases).*
